# A Glucose BioFuel Cell Implanted in Rats

**DOI:** 10.1371/journal.pone.0010476

**Published:** 2010-05-04

**Authors:** Philippe Cinquin, Chantal Gondran, Fabien Giroud, Simon Mazabrard, Aymeric Pellissier, François Boucher, Jean-Pierre Alcaraz, Karine Gorgy, François Lenouvel, Stéphane Mathé, Paolo Porcu, Serge Cosnier

**Affiliations:** 1 Laboratoire TIMC-IMAG (Techniques de l'Ingénierie Médicale et de la Complexité - Informatique, Mathématiques et Applications de Grenoble), Centre National de la Recherche Scientifique, Université Joseph Fourier, Grenoble, France; 2 DCM (Département de Chimie Moléculaire), Centre National de la Recherche Scientifique, Université Joseph Fourier, Grenoble, France; 3 Laboratoire d'Ingénierie des Systèmes Biologiques et des Procédés, Centre National de la Recherche Scientifique, Université Paul Sabatier, Institut des Sciences Appliquées de Toulouse, Toulouse, France; Massey University, New Zealand

## Abstract

Powering future generations of implanted medical devices will require cumbersome transcutaneous energy transfer or harvesting energy from the human body. No functional solution that harvests power from the body is currently available, despite attempts to use the Seebeck thermoelectric effect, vibrations or body movements. Glucose fuel cells appear more promising, since they produce electrical energy from glucose and dioxygen, two substrates present in physiological fluids. The most powerful ones, Glucose BioFuel Cells (GBFCs), are based on enzymes electrically wired by redox mediators. However, GBFCs cannot be implanted in animals, mainly because the enzymes they rely on either require low pH or are inhibited by chloride or urate anions, present in the Extra Cellular Fluid (ECF). Here we present the first functional implantable GBFC, working in the retroperitoneal space of freely moving rats. The breakthrough relies on the design of a new family of GBFCs, characterized by an innovative and simple mechanical confinement of various enzymes and redox mediators: enzymes are no longer covalently bound to the surface of the electron collectors, which enables use of a wide variety of enzymes and redox mediators, augments the quantity of active enzymes, and simplifies GBFC construction. Our most efficient GBFC was based on composite graphite discs containing glucose oxidase and ubiquinone at the anode, polyphenol oxidase (PPO) and quinone at the cathode. PPO reduces dioxygen into water, at pH 7 and in the presence of chloride ions and urates at physiological concentrations. This GBFC, with electrodes of 0.133 mL, produced a peak specific power of 24.4 µW mL^−1^, which is better than pacemakers' requirements and paves the way for the development of a new generation of implantable artificial organs, covering a wide range of medical applications.

## Introduction

Artificial implanted organs are an attractive solution to terminal failures of organs such as pancreas, urinary sphincters, kidneys or heart, but their development is thwarted by the problem of their energy supply. Sealed batteries are adequate for pacemakers [1] that consume about 10 µW, but not for more demanding applications, so that cumbersome devices are still in use, and that innovative implantable solutions are not even under research. An instance of a cumbersome device is the manual Artificial Urinary Sphincter, powered by the patient himself via a pump inserted in his scrotum, which he has to press to enable micturition. This is the only solution available today for the 10,000 new patients each year suffering from incontinence after Radical Prostatectomy [2], while a Robotized Artificial Urinary Sphincter would provide much more comfort and ease of use, but would require about 200 µW in our estimations [3]. Though Wearable Artificial Kidneys begin to be developed [4], research on Implanted Artificial Kidneys can hardly be envisaged until a permanent source of power can provide the mean 20 mW necessary for the osmotic work of kidneys in human beings. Of course, energy can be provided by transcutaneous transfer, using electro-magnetic coupling with an external source; but with the exception of cochlear implants, where a simple and miniaturized device can be used, transcutaneous energy transfer is very constraining for the patient, who accepts this only in specific cases such as artificial hearts [5], where immediate survival is at stake. Physiological constraints limit to about 100 µW the expectation of energy harvesting from Seebek thermoelectric effect, vibrations or body movements [6].

Glucose fuel cells look very promising as a source of power for implanted devices, because they could exploit glucose and dioxygen from the ECF as a source of power. Pioneering work by Drake and colleagues in the seventies raised hopes that abiotic catalysts could oxidise glucose and reduce dioxygen sufficiently efficiently to enable powering of implanted medical devices [7]. This approach had been abandoned until very recently [8], mainly because the power density was not sufficient. GBFCs exploit enzymes as catalysts, which are electrically wired by redox mediators [9]–[18]. Some GBFCs are at a pre-industrial stage and feature a substantial power output, arising a great interest as a source of “green power” for nomadic devices, while others are based on microelectrodes, exhibiting excellent ratios of power to electrode surface but low total power. Sony recently produced an instance of the former, a GBFC of 80 mL generating 100 mW under 0.30 V when fed with a glucose solution of 400 mM [19]. Unfortunately, glucose concentration in the Extra-Cellular Fluid (ECF) is only 5 mM, and the concentration of molecular oxygen, another important substrate of the GBFC, is markedly lower in blood (45 µM) than in aqueous solutions under air (200 µM). Heller and colleagues developed an instance of the latter: a micro GBFC based on bilirubin oxidase. They obtained 4.4 µW at 37°C in a physiological, glucose-enriched, buffer solution (pH 7.2, 0.14 M NaCl, 20 mM phosphate, 30 mM glucose, 0.2 mM dioxygen) [20]. The most powerful GBFC was developed by Mano [21], who increased the power density of Heller's GBFC from 90 to 280 µW cm^−2^ at low glucose concentration by using Glucose Oxidase (GOX) from *Penicillium pinophilum*, instead of the conventional GOX from *Aspergillus niger*. However, all existing GBFC, pre-industrial ones and microelectrode-based ones, use a biocathode exclusively based on bilirubin oxidase or laccase enzymes for oxygen reduction. The former requires low pH and is inhibited by chloride, while the latter is inhibited by urate anions [22]–[24], thus preventing their use in ECF (whose pH is about 7, and which contains chloride and urate anions). In order to operate a GBFC implanted in the human body, a new concept of GBFC using enzymes compatible with the characteristics of ECF has to be developed.

The ideal GBFC for operation in ECF should use enzymes and redox mediators capable of working in ECF, and be robust and easy to assemble into relatively big electrodes. We achieved this by an original mechanical confinement of the enzymes and redox mediators inside the electrodes. This enables use of several types of enzymes and redox mediators, which allowed us to select those that proved capable to work in the ECF and to produce a power compatible with the requirements of a pacemaker and potentially sufficient for powering a Robotized Artificial Urinary Sphincter.

## Results

### “Mechanically Confined” implantable GBFCs working with various enzymes and redox mediators

**Figure 1:**
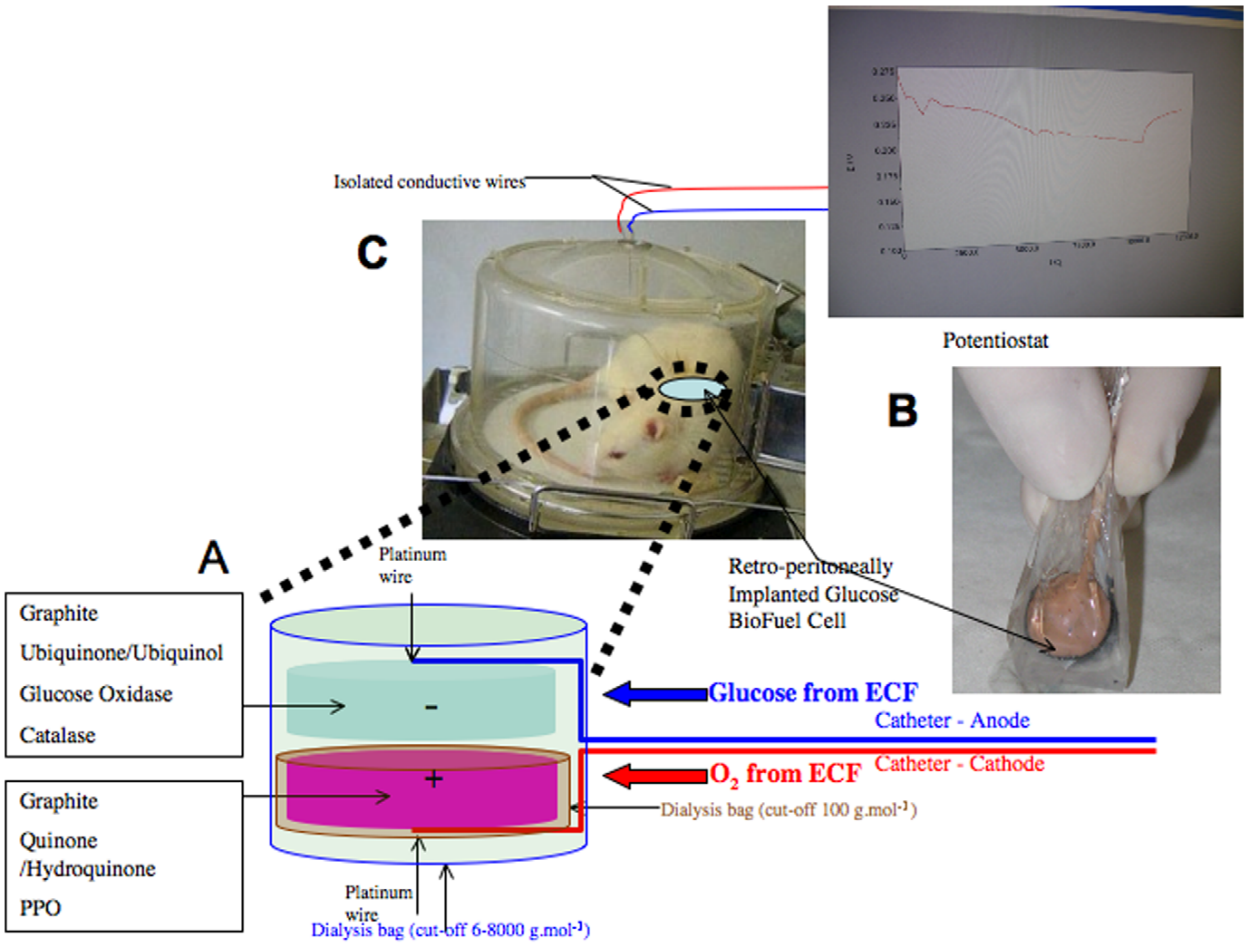
Summary of the principle, preparation, implantation and operation of an implantable “Quinone-Ubiquinone Glucose BioFuel Cell”. (A) GBFC principle. The anode is constituted of a compacted graphite disc containing ubiquinone, glucose oxidase (GOX) and catalase, while the cathode is composed of a compacted graphite disc containing quinhydrone and polyphenol oxidase (PPO). The cathode is inserted in a dialysis bag (cut-off 100 g mol^−1^), in order to prevent quinhydrone diffusion. Both electrodes are packed in an external dialysis bag (cut-off 6-8000 g mol^−1^) that lets glucose and dioxygen flow into the device. The current generated by the GBFC results from the oxidation of ubiquinol combined with the reduction of quinone. Ubiquinol and quinone are enzymatically generated by GOX and PPO respectively. (B) GBFC preparation and implantation. Each electrode measures 0.133 mL, so that the complete device can fit in the abdomen of the animal. The rat is anesthetized, a median laparotomy is performed, and the GBFC is inserted into the retroperitoneal space in left lateral position. The catheters containing the copper wires connected to the anode and cathode are subcutaneously tunnelled from the abdomen up to the back of the head of the animal, and connected to the potentiostat. Finally, the muscular abdominal wall and the skin are separately sutured and the animal is allowed to recover from anesthesia. (C) GBFC operation. 4 hours after implantation, cycles of discharge (at constant current of 10 µA) and of charge are recorded via a potentiostat until sacrifice of the animal.

In contrast to current GBFCs, where enzymes and redox mediators are covalently bound to the electrode, we mechanically confined the contents of the electrodes, by use of dialysis membranes and/or mechanical compression of graphite particles, enzymes and redox mediators (*Materials and Methods*). This process, summarized in Figure 1, required simple procedures involving classical chemicals and materials, and allowed use of soluble (quinone, hydroquinone) or poorly-soluble (ubiquinone, ubiquinol) redox mediators, and of different enzymes (GOX at the anode, PPO or urease at the cathode). One of these GBFCs used as fuel not only glucose, but also urea.

### *In vitro* demonstration of performance of our “Mechanically Confined” Quinone-Ubiquinone GBFC with GOX and PPO

**Figure 2:**
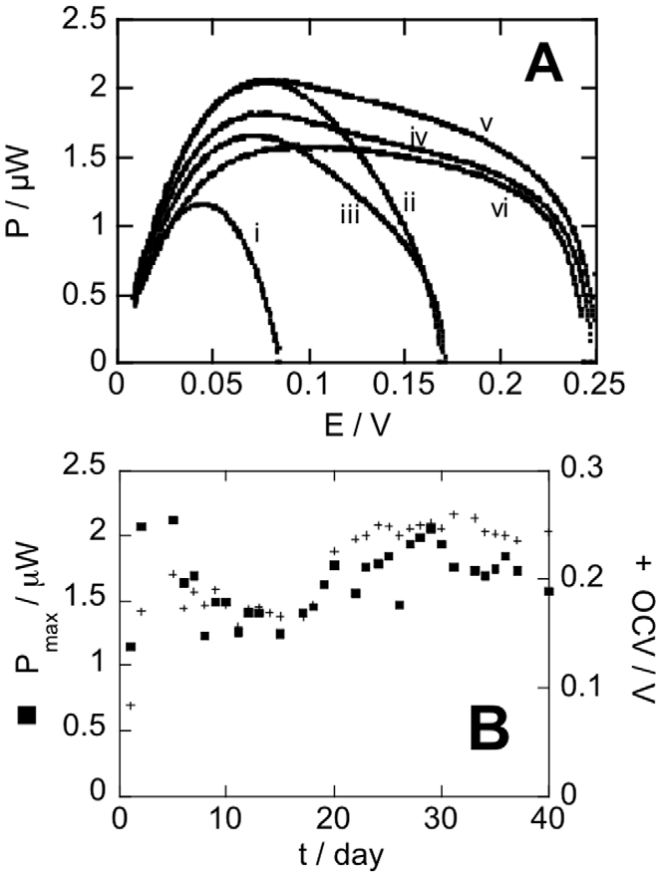
In vitro performances of a Quinone-Ubiquinone GBFC. The GBFC is immersed in 50 mL HEPES buffer (2.5 10^−2^ mol L^−1^; pH 7.2) containing 5.5 10^−3^ mol L^−1^ glucose and 0.15 mol L^−1^ NaCl. (A) Power-voltage profile; i) first day, ii) second day, iii) sixth day, iv) twenty fourth day, v) twenty ninth day vi) fortieth day. (B) Maximum power (▪) and OCV (+) as a function of time.

We experimented *in vitro* our Quinone-Ubiquinone GBFC, under a concentration of glucose similar to that of ECF (5.5 10^−3^ mol L^−1^ glucose), and with a phosphate buffer of 2.5 10^−2^ mol L^−1^ yielding a pH of 7.2 (thus simulating the pH of ECF, which is controlled by a very efficient bicarbonate buffer), the two electrodes being wrapped in the expanded PolyTetraFluroroEthylene (exPTFE) membrane that was used later for *in vivo* experiments. The operational stability of this GBFC was evaluated for a constant current of 10 µA in these conditions. The voltage and hence the cell power decreased to ca. 9% (0.15 µW) of the initial value over 1 hr, and then the GBFC maintained a quasi-constant power (1.47 µW), delivering 10 µA during 25 hrs. To demonstrate the stability of the GBFC, we recorded its performances for 40 days (Fig. 2). Open circuit voltage (OCV) was continuously monitored, while power-voltage profile and discharge at constant current (5 µA) for 10 min were daily recorded. The performances of the GBFC (power, open circuit voltage OCV and discharge) first increased from the first to the second day. The average maximum power from 30 to 40 days was about 1.65 µW (standard deviation 0.13 µW), reflecting an excellent operational stability. The performance of the GBFC was not diminished over time, since the OCV reached 250 mV during the last two weeks, discharge curves keeping the same shape.

### *In vivo* demonstration of performance of our Quinone-Ubiquinone GBFC

**Figure 3:**
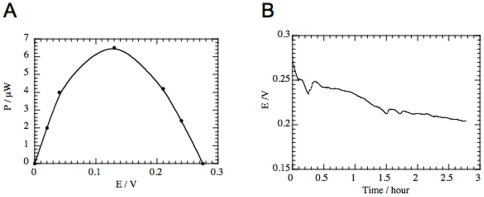
Performance of a “Quinone-Ubiquinone GBFC” implanted in a male Wistar rat (514 g weight). (A) Power-voltage profile. A peak power of 6.5 µW is observed at 0.13 V, yielding a maximum specific power of 24.4 µW mL^−1^. (B) Discharge curve. Discharge of the GBFC starts 4 hours after implantation. The chronopotentiometry was recorded for a constant current (10 µA). A stable production of more than 2 µW is observed for several hours.

Having shown *in vitro* power generation by our Quinone-Ubiquinone GBFC, we next ascertained whether it was able to work when implanted in an animal. A male Wistar rat (514 g weight) was anesthetized and this GFBC was surgically inserted into its retroperitoneal space, enabling glucose and O_2_ from the ECF to flow into the GBFC. After the rat had recovered from anesthesia and was allowed unrestricted movement, cell performance was evaluated (Fig. 3). The OCV of the GBFC was 0.275 V while the maximum power was 6.5 µW (at 0.13 V), yielding a maximum specific power of 24.4 µW mL^−1^. The voltage decreased from 0.27 to 0.22 V during the first 80 min and was then quasi-stable, with a slow decrease of 9 mV h^−1^. These experiments confirmed the capacity of a GBFC to work in ECF and to produce 2 µW (7.52 µW mL^−1^) for several hours.

### Demonstration of performance over an extended period of time

**Figure 4:**
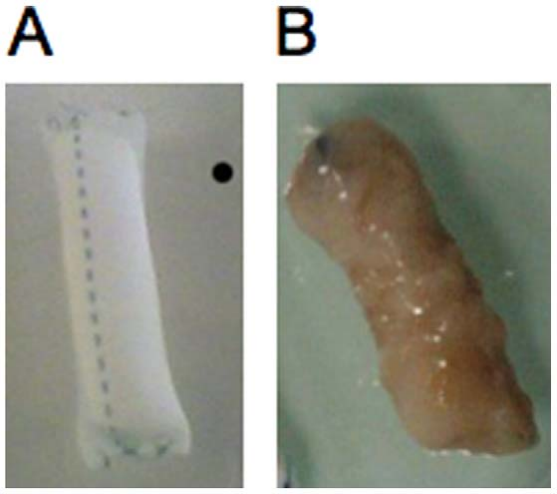
Implants containing both Glucose Oxidase and catalase, before and after implantation in a rat. Implants containing both GOX and catalase, immobilized on barium alginate beads, in dialysis tubing wrapped in an exPTFE coating. (A) Before implantation. (B) After 3 months of implantation. A neo-vascular network can be seen, no sign of inflammation is present, proving the good tolerance of the rat for the implant.

In order to power implanted organs, a GBFC must prove over an extended period of time that it can remain functional, and that it can extract sufficient glucose and O_2_ from the ECF. We carried out stability experiments consisting in daily recording the power-voltage profile (during discharges at 5 µA for 10 min) for an implanted GBFC. As previously observed, after an initial increase, the performances (power 1.8 µW and OCV 200 mV) for a smaller rat (444 g weight) remained stable (standard deviation 0.17 µW) until sacrifice of the animal after 11 days. Regarding long term glucose and O_2_ extraction from the ECF, we implanted in the retroperitoneal space of a rat a dialysis tubing of 4 mL wrapped in an exPTFE coating, containing GOX and catalase, and monitored during 3 months the production of gluconate in the daily urines of the animal (*Materials and Methods S1*). At sacrifice, no sign of inflammatory reaction against the implant was observed, while a neo-vascular network had developed around the implant (Fig. 4). A mean daily production of 555 µmoles day^−1^ of gluconate was measured.

### *In vitro* and *in vivo* demonstration of performance of “Mechanically Confined” Quinhydrone pH-based Glucose and Urea BioFuel Cell working with GOX and urease

**Figure 5:**
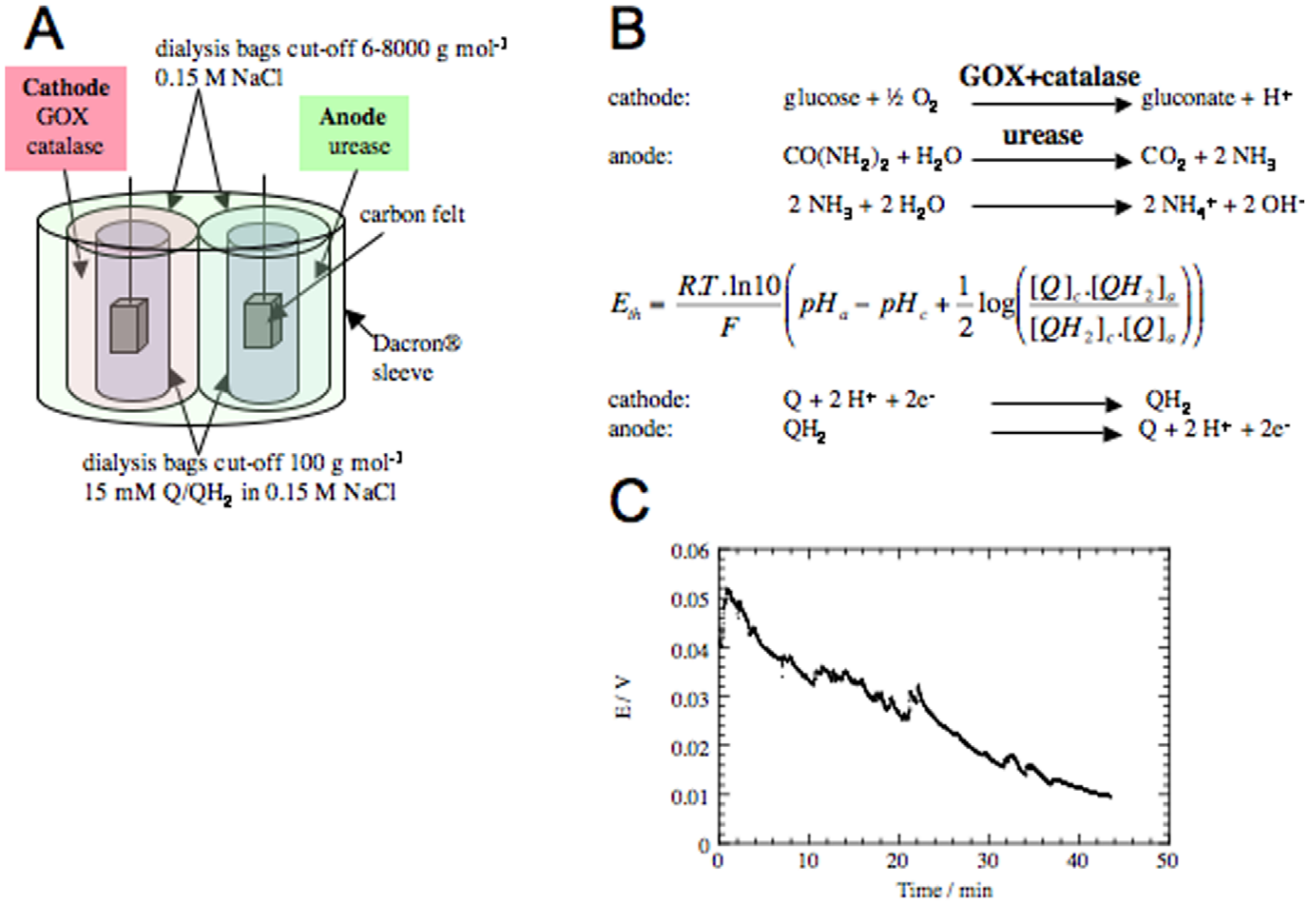
Implantable “Quinhydrone pH-based Glucose and Urea BioFuel Cell” with mechanically confined electrodes. (A) Schematic representation. In each electrode, the redox species, quinone (Q) and hydroquinone (QH2) are confined close to a carbon felt by a first dialysis bag (nominal cut-off of 100 g mol^−1^). This bag separates the redox species from the enzymes contained in a second dialysis bag with a nominal cut-off of 6–8000 g mol^−1^. This second dialysis bag contains GOX and catalase for the anode, and urease for the cathode. The two electrodes are packed together in a Dacron® sleeve. (B) Electro-chemical reactions at the electrodes. Action of the GOX at the cathode locally decreases the pH, while action of the urease at the anode locally increases pH. Nernst law governs the difference of potential between anode and cathode (subscripts *a* is used in this equation to identify pH and concentrations of species at the anode, subscript *c* denoting the cathode). At the cathode, quinone (Q) is reduced into hydroquinone (QH_2_), while at the anode hydroquinone (QH_2_) is oxidised into quinone (Q). (C) Discharge curve under 100 nA. This curve was recorded after implantation in the retroperitoneal space of a rat, for a constant current of 100 nA. It corresponds to a mean power of 3 nW during 45 minutes.

In order to demonstrate that the concept of “mechanical confinement” allows use of several types of enzymes and redox mediators, we developed and tested another instance of a “Mechanically Confined” GBFC, based on the dependence on pH of the potential of quinhydrone, an equimolar mixture of quinone (Q) and hydroquinone (QH_2_). The principle of this BioFuel Cell, which uses as fuel both glucose and urea and is detailed in *Materials and Methods*, is summarized in Figure 5. An *in vitro* demonstrator showed that this principle led to a ΔpH of 4.8 generating under 10 µA a potential difference of 265 mV, corresponding to a maximum power of 2.65 µW. Power of 3 nW was obtained during 45 min after implantation in the retroperitoneal space of a rat (Fig. 5), illustrating the viability of this concept *in vivo*.

## Discussion

These experiments are the first ones reporting successful operation of a GBFC inside an animal. This was made possible by an innovative reduction of O_2_ into water by PPO, an enzyme capable to work efficiently in the specific conditions of the Extra-Cellular Fluid, which is not the case of enzymes such as laccase or bilirubin oxidase, classically used in GBFCs. Use of this enzyme was facilitated by the application of a very simple process of construction of the electrodes, based on mechanical confinement of redox mediators and of enzymes, which provides a cheap and simple alternative to the classical electric wiring obtained by covalent binding of these elements on an electron collector. This process allows use of virtually any type of enzymes and redox mediators that would be capable to work in the ECF. We proved that pH-based BioFuel Cells exploiting as fuel not only glucose, but also urea, could work. Though the enzymes we used for this pH-based BioFuel Cell work in ECF conditions, the potential of this specific approach seems today limited to *in vitro* applications, until a solution is found to isolate the GBFC from the bicarbonate buffer of the ECF. Indeed, the difference of power between *in vitro* and *in vivo* experiments suggests that this very efficient buffer (about 25 mM) prevents *in vivo* application of this specific approach.

We obtained a stable *in vivo* production of 2 µW in a device of some mL whose electrodes are 0.133 mL. This power may look limited, when compared to the 4.4 µW obtained by Heller and colleagues [20]. However, the major difference lies in the fact that our performances were recorded with a device implanted inside the ECF of a rat, while the results reported by Heller and colleagues were obtained *in vitro*. Besides, in the latter experiment, the solution was enriched in glucose (30 mM glucose) and under air (so that the concentration of dioxygen was 200 µM), which are significantly different conditions with respect to those of ECF (where glucose is below the 5 mM of blood glucose level and dioxygen below the 45 µM of venous level).

An important issue will be to demonstrate that our GBFC can work during months or years when implanted in animals. We demonstrated that an implant with GOX and catalase remained operational during 3 months, and kept capable to produce about 100 times more gluconate than what our GBFC did to produce 2 µW. Indeed, according to Faraday's law, since in our GBFC each mole of glucose provides two moles of electrons, our GBFC consumes each day 4.5 µmoles of glucose, in order to produce 2 µW under 200 mV. This figure should be compared to the daily consumption of 555 µmoles of glucose reported in section 2.4 with a 4 mL device containing GOX. This experiment tends to prove that extraction of glucose from ECF will not be the limiting factor to improve the performance of our GBFC. The fact that GOX remained operational during 3 months is very encouraging, and is corroborated by experiments reported by Minteer [25], who proved that implanted enzymes could be kept fully operational during more than one year, in conditions similar to the ones requested by our GBFC concept.

With its present design, our GBFC is capable to produce a peak power of 24.4 µW mL^−1^, and a stable power of more than 7.52 µW mL^−1^. This is already significant with respect to the requirements of medical devices (typically 10 µW for a pacemaker [1]). Though sealed batteries are perfectly adequate for pacemakers (they have to be surgically replaced after 5 to 8 years, which is quite acceptable), their use in applications requesting 200 µW or more would require significant increase in the performance they reach today in clinical practice (since they would have to be replaced about twice a year). It should be noted that early pacemakers had volumes up to 90 mL^1^, which shows that implanted devices of such a big volume can be accepted, provided that there is a significant medical added-value. With our present level of stable performance, we could expect to generate in an animal such as a pig about 1 mW with a battery of about 133 mL. We are beginning to explore the possibility to use this approach to power a Robotized Artificial Urinary Sphincter, which requires in our estimations about 200 µW [3], corresponding to a GBFC similar to the ones we tested, but of about 26 mL. This is about the size of the balloon that is implanted today in the abdomen of patients to whom manually-controlled Artificial Urinary Sphincters are proposed [2]. We also expect significant improvement from the use of other enzymes and redox mediators. Indeed, the concept of mechanical confinement enables straightforward integration of any such enzyme or redox mediator, so that it is not unrealistic to get with GBFCs exploiting the concepts we described to produce tens of mW or more, thus opening possibilities for research on new generations of implanted medical devices.

## Materials and Methods

### Ethics Statements

The care of the rats was approved by the European Communities Council Directive Animal Care and Use Committee and performed in accordance to their guiding principles (European Communities Council Directive L358-86/609/EEC). All protocols involving living animals were performed under license from the French Ministry of Agriculture (License number 38018).

### “Mechanically Confined” Quinone-Ubiquinone GBFC with GOX and PPO

Mechanical compression of graphite particles, enzymes and redox mediators provided mechanically stable composite discs of 0.133 mL, and allowed the coimmobilisation of poorly soluble redox mediators such as coenzyme Q10 or ubiquinone, and hence a non-covalent electric wiring (*Materials and Methods S1*). A platinum wire fixed on one side of the disc by a conductive adhesive connected each composite graphite disc. A schematic representation of our process is outlined in Figure 1. The anode contained a mixture of graphite, ubiquinone, glucose oxidase (GOX) and catalase. The cathode contained quinhydrone, combined with polyphenol oxidase (PPO) and graphite, and was inserted in a cellulose acetate dialysis bag with a nominal cut-off of 100 g mol^−1^ to prevent diffusion of quinhydrone. The two electrode discs were placed face to face, with the platinum wire outside, and then inserted in an external dialysis bag with a nominal cut-off of 6-8000 g mol^−1^. Each isolated platinum wire was inserted in a catheter onto which the external dialysis bag was sealed. The external dialysis bag prevented any diffusion of enzymes. Glucose and O_2_ from the outside flowed into the device across the external dialysis bag. In presence of glucose, GOX generated ubiquinol from ubiquinone while catalase eliminated H_2_O_2_ produced by the side reaction of dioxygen with GOX. In the presence of dioxygen, PPO catalysed the oxidation of phenols and di-phenols into quinoid products, dioxygen being reduced into water. Thus, at the cathode PPO regenerated the quinone form reduced previously into hydroquinone by the battery reaction. In contrast to laccase and bilirubin oxidase, PPO could efficiently operate at pH 7 and was not inhibited by products present in physiological fluids. The circuit was then closed with a potentiostat enabling automatic external resistance adaptation in order to keep a constant current in the circuit.

### “Mechanically Confined” Quinhydrone pH-based Glucose and Urea BioFuel Cell working with GOX and urease

Each electrode comprised a carbon felt inserted in a first dialysis bag with a nominal cut-off of 100 g mol^−1^, initially containing quinhydrone (*Materials and Methods S1*). Each bag was then inserted in a second dialysis bag with a nominal cut-off of 6–8000 g mol^−1^, containing GOX and catalase for the anode, and urease for the cathode. The two electrodes were packed together in an exPTFE membrane. In the presence of O_2_, GOX and catalase catalyze the production of gluconate and protons at the cathode, while urease activity on urea creates hydroxyl ions at the anode. According to the Nernst equation, the gradient of pH between the two electrodes, which contain the same couple of pH-sensitive redox mediators, modifies the electrical potential of each electrode and generates electron exchanges.

### Materials and Methods S1

Detailed presentations of the following materials and methods are described in the separate “Materials and Methods S1” file.

## Supporting Information

Materials and Methods S1 Supplementary Materials and Methods for A Glucose BioFuel Cell Implanted in Rats.
(0.07 MB DOC)
