# Supplementary material for: A Glucose BioFuel Cell Implanted in Rats
Source: PLoS One. 2010 May 4;5(5):e10476. doi: 10.1371/journal.pone.0010476 (PMC2864295; doi:10.1371/journal.pone.0010476)
Supplement: Materials and Methods S1 — Supplementary Materials and Methods for A Glucose BioFuel Cell Implanted in Rats. (0.07 MB DOC) [file pone.0010476.s001.doc]

Materials and Methods S1

Supplementary Materials and Methods for *A Glucose BioFuel Cell Implanted in Rats*

Philippe Cinquin^1^*, Chantal Gondran^2^, Fabien Giroud^2^, Simon Mazabrard^2^, Aymeric Pellissier^2^, François Boucher^1^, Jean-Pierre Alcaraz^1^, Karine Gorgy^2^, François Lenouvel^1^, Stéphane Mathé^3^, Paolo Porcu^1^, Serge Cosnier^2^*

## Chemicals:

Glucose oxidase (GOX) (EC 1.1.3.4 type VII, from *Aspergillus Niger,* 179 U mg^-1^), Catalase (EC 1.11.1.6, from *Bovine Liver,* 1610 U mg^-1^), Urease (EC 3.5.1.5, type IX, from *Jack Beans*, 70.4 U mg^-1^), coenzyme Q10 (ubiquinone UQ, 98 %), quinhydrone (equimolar mixture of quinone - Q – and of hydroquinone - QH_2_) and graphite particle were purchased from Sigma-Aldrich. Polyphenol oxidase (PPO) (EC 1.14.18.1, from *mushroom*, 5244 U mg^-1^) was purchased from Fluka.

## Instrumentation:

Electrochemical experiments were performed using an Autolab potentiostat 100 (Eco Chemie, Utrecht, The Netherlands), using biocathode as the working electrode and bioanode as the counter electrode. Carbon felts (RVC2000®) were supplied by Le Carbone Lorraine. Dialysis membranes were purchased from Spectrumlabs: Spectra/Por® Dialysis membrane, MWCO 6-8000 g mol^-1^, flat width 32 mm, diameter 20.4 mm, vol/length 3.3 mL/cm; Spectra/Por® CE, cellulose ester membrane, MWCO 100 g mol^-1^, flat width 31 mm, diameter 20 mm, vol/length 3.1 mL/cm.

## “Mechanically Confined Quinone-Ubiquinone GBFC working with GOX and PPO”: Electrodes fabrication

The bioanode was prepared as follows: graphite particles (350 mg), 110 mg of UQ, 0.5 mL water and glycerol (50 μL) were thoroughly mixed in ceramic mortar. Then, 7 mg GOX and 3 mg catalase, solubilised in 100 μL water, were incorporated to 400 mg of the preceding mixture and thoroughly hand-mixed. The biocathode was similarly fabricated: graphite particles (350 mg), 170 mg of quinhydrone, 0.3 mL water and glycerol (25 μL) were thoroughly mixed in ceramic mortar. Then, 4.5 mg PPO, solubilised in 100 μL water, were incorporated to 400 mg of the preceding mixture and thoroughly hand-mixed. The resulting enzymes-graphite-redox mediator paste was pressed at 10 000 kg cm^-2^ to form a disc. The surface area and thickness of disc are 1.33 cm² and 0.1 cm respectively. A platinum wire was fixed by a conductive carbon glue on one side of the disc, which was then covered by silicon film to reinforce the mechanical strength of the biocoating and electrical contact.

## “Mechanically Confined Quinone-Ubiquinone GBFC working with GOX and PPO”: Electrochemical material preparation

To elaborate the GBFC, the cathode disc was first soaked into 0.15 M NaCl solution and enclosed in dialysis bag with a molecular weight cut-off at 100 g mol^-1^. Then, the anode disc and the bag containing the cathode disc were each confined into a dialysis bag with a molecular weight cut-off at 6-8000 g mol^-1^, containing 0.15 M NaCl. The opening of dialysis membranes was sealed by silicone. The platinum wires were welded to the copper wires (diameter 0.25 mm) contained in catheters (length 0.80 m, diameter 0.58 mm). The dialysis membranes, solutions and catheters were sterilized by autoclaving (121 °C, 20 minutes), in physiological isotonic solution.

The GBFC is recharged through enzymatic reactions:

cathode: QH_2_ + ½ O_2_
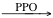
 Q + H_2_O

anode: glucose + UQ
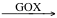
 gluconolactone + UQH_2_

The GBFC will discharge according to the following reactions:

cathode: Q + 2 H^+^ + 2e^-^🡪 QH_2_ E°' = 280 mV vs SHE

anode: UQH_2_🡪 UQ + 2 H^+^ + 2e^-^ E°' = 110 mV vs SHE

## “Mechanically Confined Quinone-Ubiquinone GBFC working with GOX and PPO”: in vitro tests

The buffering effect of ECF in the body on GBFC functioning was investigated in vitro by performing biofuel cell assays in 25 mM phosphate buffer (pH = 7.2, mimicking the effect of the carbonate buffer in the body) containing 10 mM glucose and 0.15 M NaCl.

## “Mechanically Confined Quinone-Ubiquinone GBFC working with GOX and PPO”: in vivo tests

A “Quinone-Ubiquinone GBFC” was implanted on two rats (weight 514 g and 512 g), according to the procedure described below (*surgical procedures on rats*). After stabilization of the open circuit potential, cycles of discharge and charge were performed during a period of 12 hours, voltage being monitored. Then, the rat was sacrificed. Discharge currents of 10 μA and 50 μA were tested via the potentiostat.

## In vitro demonstrator for “Mechanically Confined Quinhydrone pH-based Glucose and Urea BioFuel Cell working with GOX and urease”

Both electrodes were composed of carbon felt (1.2 × 0.7 × 0.3 cm^3^) immersed in an aqueous 0.15 M NaCl solution (1 mL) containing quinhydrone(3.2 mM) and enclosed in a dialysis membrane with a molecular weight cut-off of 100 g mol^-1^. A fuel cell (Supplementary Figure 1) was elaborated with a device constituted by two compartments (7 mL each) separated by a hydrogel wall (agar-agar; thickness: 4 mm). The anode and the cathode were immersed in 0.15 M NaCl containing 5.0 mM D-glucose and 3.5 mM urea. Beside the electrolyte and substrates, the anodic compartment contained urease (5.2 mg) while the cathodic compartment contained GOX (2 mg) and catalase (0.8 mg). In the cathodic compartment, the presence of catalase prevents the accumulation of H_2_O_2_ that can deactivate the GOX enzymes. H_2_O_2_ was produced by a side reaction of O_2_ with GOX in the presence of glucose, the reduced form of GOX (FADH_2_) being re-oxidized into GOX (FAD) by O_2_. Catalase thus catalyzes the dismutation of H_2_O_2_ into H_2_O and O_2_.

As a consequence of the following enzymatic reactions, a pH gradient is biologically created:

cathode: glucose + ½ O_2_
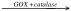
 gluconate + H^+^

anode: CO(NH_2_)_2_ + H_2_O
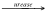
 CO_2_ + 2 NH_3_

2 NH_3_ + 2 H_2_O 2 NH_4_^+^ + 2 OH^-^

The electromotive force of the pH-based GBFC is given by:


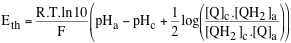


where subscript *a* identifies anode pH and concentrations, subscript *c* corresponding to the cathode.

For identical concentrations of the redox species, the difference in pH provides a potential difference between the cathode and the anode.

The pH-based BFC will discharge according to the following reactions:

cathode: Q + 2 H^+^ + 2e^-^🡪 QH_2_

anode: QH_2_🡪 Q + 2 H^+^ + 2e^-^

## In vivo “Mechanically confined Quinhydrone pH-based Glucose and Urea BioFuel Cell working with GOX and urease”: electrode fabrication and electrochemical material preparation

Both electrodes are composed of carbon felt (0.8 × 0.6 × 0.4 cm^3^) immersed in an aqueous 15 mM quinhydrone solution (1 mL) and enclosed in a dialysis membrane with a molecular weight cut-off of 100 g mol^-1^ (Figure 2). Electrodesare welded to copper wires (diameter 0.25 mm) contained in catheters (length 0.80 m, diameter 0.58 mm). Each dialysis bag (anode and cathode) was placed in another dialysis bag with a molecular weight cut-off of 6-8000 g mol^-1^ containing 0.15 M NaCl (2 mL) and urease (114 mg) for the anode and GOX (44.7 mg) and catalase (7.5 mg) for the cathode. The opening of dialysis membranes was sealed by silicone. The dialysis membrane, solutions and catheters were sterilized in physiological solution. The two compartments are incorporated into Dacron® sleeve that was sterilised by autoclaving (121 °C, 20 minutes).

## In vivo “Mechanically Confined Quinhydrone pH-based Glucose and Urea BioFuel Cell working with GOX and urease”: in vivo tests

A “Quinhydrone pH-based Glucose and Urea BioFuel Cell” was implanted on one rat (weight 460 g), according to the procedure described below (*surgical procedures on rats*). After stabilization of open circuit potential, cycles of discharge and charge were performed during a period of 12 hours, voltage being monitored, then the rat was sacrificed. Discharge currents of 100 nA and 1 μA were tested via the potentiostat.

## Demonstration of long-term in-vivo efficiency of enzymes stabilized in a barium-alginate gel

A barium-alginate gel was elaborated as follows. 1) Both, GOX (240 mg) and catalase (30 mg) were mixed to 50 mL of a 1 % sodium alginate solution. 2) Droplets of the latter were dropped in a 200 mL stirred 0.1 M barium chloride solution (300 rpm) using a Gilson peristaltic pump and a 1.6 mm tubing The flow was set at 150 rpm on the pump. Height of dropping was 10 cm. The gelation time was 40 minutes under constant stirring. The beads were washed twice with distilled water. The obtained gel beads were introduced in a dialysis tubing (diameter 15 mm, MWCO 12-14000 gmol^-1^) that was closed at both ends by a node. The volume of the obtained closed bag was measured to 4 mL (14 cm^2^ in surface), corresponding to a quantity of GOX of 1040 Units (38 beads were inserted in the dialysis bag; each bead is 50 µL big, and contains 27 units of GOX). The bag was then wrapped into a piece of 0.8 cm of diameter and 5 cm of length of an endovascular prosthesis (VXT ADVANTA), an expanded PolyTetraFluroroEthylene membrane (exPTFE). The exPTFE membrane was sewed at each end with a non-resorbing suture performed with a 6.0 thread. The implant was inserted into a rat weighing 360 g with the procedure described below (*surgical procedures on rats*). The rat was installed in a metabolic cage, and allowed to live for 3 months. On a daily basis, body weight was monitored and urine was collected for volume measurement and sampling, allowing estimation of daily production of gluconate and gluconolactone. The respective amounts of these specific and non-metabolisable products of GOX action on glucose were determined by High Performance Anion Exchange Chromatography HAEPC with a Decade detector equipped with a Carbopac PA10 column (Dionex). A similar procedure was applied to a test rat, which received an implant where no enzymes had been immobilized. After sacrifice, the implant was visually cleaned and observed.

## Surgical procedures on rats

Male Wistar rats weighing 300 – 560 g were anesthetized with sodium pentobarbital (50 mg/kg, i.p.). A median laparotomy was performed and the GBFC or one of the implants was inserted into the retroperitoneal space in left lateral position. For GBFC procedures, the catheters containing the copper wires were subcutaneously tunnelled from the abdomen up to the back of the head of the animal. The muscular abdominal wall and the skin were finally separately sutured and the animals were allowed to recover from anaesthesia. After surgery, the animals received a single injection of an analgesic (Rimadyl, 5 mg kg^-1^, i.m.). The rats were sacrificed under anaesthesia (sodium pentobarbital 50 mg kg^-1^, i.p.), by intra-cardiac injection of sodium pentobarbital (100 mg). The care of the rats was approved by the European Communities Council Directive Animal Care and Use Committee and performed in accordance to their guiding principles (European Communities Council Directive L358-86/609/EEC). All protocols involving living animals were performed under license from the French Ministry of Agriculture (License number 38018).
